# Rituximab Administration and Reactivation of HBV

**DOI:** 10.1155/2010/182067

**Published:** 2010-12-01

**Authors:** Yutaka Tsutsumi, Reiki Ogasawara, Yusuke Kamihara, Shinichi Ito, Yoshiya Yamamoto, Junji Tanaka, Masahiro Asaka, Masahiro Imamura

**Affiliations:** ^1^Department of Hematology, Hakodate Municipal Hospital, Hakodate 041-8680, Japan; ^2^Department of Internal Medicine, Hakodate Municipal Hospital, 1-10-1 Minato-Cho, Hakodate 041-8680, Japan; ^3^Department of Gastroenterology, Hakodate Municipal Hospital, Hakodate 041-8680, Japan; ^4^Department of Hematology and Oncology, Hokkaido University Graduate School of Medicine, Sapporo 060-8638, Japan; ^5^Department of Gastroentelology, Hokkaido University Graduate School of Medicine, Sapporo 060-8638, Japan

## Abstract

Rituximab is a drug used for the treatment of B-cell non-Hodgkin's lymphoma, and its range of use has expanded to the treatment of collagen diseases such as idiopathic thrombocytopenic purpura and rheumatoid arthritis. One serious complication of rituximab use is the reactivation of dormant hepatitis B virus, and prevention of this phenomenon has become an urgent issue. This paper provides a general outline of the problem through an analysis of patient cases that we and other groups have experienced to date.

## 1. Introduction

Rituximab is a human-mouse chimeric monoclonal antibody that targets the B-cell CD20 cell surface protein and has become indispensible for the treatment of B-cell non-Hodgkin's lymphoma [1–3]. however, Complications in the form of severe viral infections have been observed, and methods to counter these infections are presently being evaluated [4–7]. Reactivation of hepatitis B virus (HBV) is one such complication. Prior to the introduction of rituximab, reactivation of HBV was a major complication of chemotherapy-induced immunosuppression [8–20]. The events leading to HBV reactivation during rituximab/chemotherapy combination treatment have been reported by various groups, as have the effects of preventive administration of the nucleoside analogue lamivudine on the suppression of HBV reactivation [21–33]. However, a number of issues remain to be determined, such as the level of HBV resistance to lamivudine, the optimal lamivudine administration period, and even whether or not lamivudine is the best preventive drug to use. HBV reactivation may result in a number of serious outcomes, including death from hepatitis [23–37]. Additionally, even in the event that hepatitis is prevented, possible problems in subsequent lymphoma treatment or in anticipated outcomes may arise, such as lymphoma recurrence or shortened survival prognosis. In this paper based on studies from various groups, we explored issues in preventive nucleoside analog administration, including optimal administration period and the utility of concurrent use of these drugs. The discussion includes a compilation of our results on preventive administration of nucleoside analogs in HBs antigen- (HBsAg-) positive cases, which were accrued from the debut of rituximab in Japan in 2002 to December 2008.

## 2. HBV Reactivation during Rituximab Administration

Acute hepatitis caused by HBV is initially suppressed by cytokines secreted from NK and other types of cells. CD8-positive cells subsequently induce a CTL reaction which then eventually leads to hepatitis. Since hepatitis is triggered by CTLs, a time lag exists from initial infection to hepatitis onset [38, 39]. However, hepatitis that arises from HBV reactivation, unlike that which occurs during conventional HBV infection, is under a state of immunosuppression of the normal immunological responses to HBV, leading to increased viral replication and widespread infection of hepatocytes. When immunosuppression is removed by discontinuation of chemotherapy, immune competence is restored and infected hepatocytes are rapidly destroyed, leading to hepatitis. It is therefore likely that reactivated HBV results in a shorter time period to hepatitis progression than seen in conventional HBV infection. We believe that this mechanism of hepatitis aggravation can explain those case patients who die despite administration of lamivudine in response to HBV reactivation during chemotherapy or use of immunosuppressive drugs. In contrast, HCV tends to become chronic through suppression of the induction and amplification of immune responses (such as interferons) from the outset, and this may be why hepatitis caused by HCV reactivation during the use of anticancer or immunosuppressive drugs is less likely to become severe [40–42]. 

We have reported on reactivation of HBV and hepatitis during rituximab treatment alone or in combination with chemotherapy. Other groups have reported a rate of HBV reactivation of 20%~55% [15, 18–20]. One report claims an HBV reactivation rate of 3%, even in HBsAg-negative cases [43]. Umemura et al. reported a 4% rate of HBV reactivation in HBsAg-positive patients based on a questionnaire conducted in Japan, which is lower than previous studies, and 20% of these patients developed fatal hepatitis [44]. Frequent HBV reactivation is also thought to occur as a complication of chemotherapy during treatment of lymphomas and may be influenced by steroids [18, 45, 46]. Since the initial use of rituximab in the clinic, debate has centered on whether rituximab alone can induce HBV reactivation or whether it does so only when used in combination with chemotherapy. Initially, we believed that rituximab alone was unlikely to induce HBV reactivation [21]. However, subsequent reports by us and by Yang et al. have shown that HBV reactivation can occur with rituximab alone, and it is therefore likely that rituximab itself can induce HBV reactivation [22, 37]. Although reactivation of HBV is deemed more likely when rituximab is combined with chemotherapy or steroid therapy, clearly the use of rituximab alone cannot be deemed completely safe [22]. Future prospective analyses are needed to definitively answer whether or not reactivation of HBV is more prevalent in patients given rituximab alone or given chemotherapy without rituximab. Nonetheless, in a questionnaire survey at a Hokkaido facility examining and treating blood diseases, HBV reactivation cases were observed only in patients given rituximab alone. These results support the study by Yeo et al., showing that reactivation of HBV is more prevalent in patients given rituximab alone compared with chemotherapy alone [22, 47].

## 3. Risk Factors for HBV Reactivation

To date, various investigators have identified a number of risk factors for HBV reactivation which include being male, HBs antigen positivity, HBV-DNA level, presence of lymphoma, use of anthracyclines or steroids, second- and third-line anticancer drug treatment, and youth. A review of these factors by Yeo et al. showed that youth, being male, and liver function prior to chemotherapy were potential risk factors. Furthermore, Yeo et al. conducted an analysis on HBV reactivation in patients using rituximab, as there is a potential for risk factors to change in such cases. Including risk factors which had previously been reported, they list being male, lacking HBs antibodies, and using rituximab as additional risk factors. Further analyses on such risk factors are needed in order to enable the stratification of HBV-infected patients receiving rituximab in order to identify those that require preventive administration with nucleoside analogs [20, 47, 48].

## 4. HBsAg-Positive, Anti-HBc-Positive, and HBV-DNA-Positive Cases

In HBsAg-positive patients, there have been reports of HBV reactivation during chemotherapy [8–20]. Increased liver function and a tendency for increased jaundice and lowered albumin have also been reported in hepatitis cases caused by HBV reactivation, compared to primary HBV infection [44]. Similarly, HBV reactivation has been reported in patients receiving chemotherapy in addition to rituximab [21–37]. For these patients, attempts to prevent HBV reactivation are needed regardless of whether or not rituximab is given. Preventive administration of nucleoside analogs is currently recommended for such patients, and chemotherapy under preventive administration is desirable [49–63]. Although there are few complete reports of cases in which rituximab was used in combination with preventive treatments, He et al. among others have suggested the effectiveness of preventive lamivudine [49, 64, 65]. Loomba et al. reported that preventive lamivudine therapy reduced HBV-reactivation-related hepatitis by 79% or more, based on an analysis of previous studies [49]. However, it has been reported that with lamivudine administration, drug resistance increases yearly by 15%–20% [66, 67]. Thus, with preventative administration of lamivudine, the emergence of resistant HBV strains is highly problematic.

A report by Pelizzari et al. suggested that lamivudine administration may result in a lower likelihood of emergent drug resistance compared to treatments for conventional hepatitis B [56]. However, Picardi et al. reported numerous instances of HBV genome mutations in patients receiving chemotherapy with fludarabine [68]. In addition, they have shown that there is a high risk of HBV resistance to lamivudine in patients undergoing treatments that have powerful immunosuppressive effects. A similar report exists for chemotherapy combined with rituximab, and it is possible that similar occurrences may be observed when steroids or fludarabine are used together with rituximab [69]. From these observations, as well as considering issues of emergent drug resistance, administration of entecavir is preferred over lamivudine for the prevention of HBV reactivation. Additionally, even among HBsAg-positive cases, preventive treatment may change in relation to the level of HBV-DNA present. Various guidelines recommend that when more than 1 year of long-term nucleoside analog administration against HBV-DNA is necessary, the analog should be changed to entecavir. In patients who have a high level of HBV-DNA, it is desirable to use drugs such as entecavir, based on its effectiveness against the YMDD mutation [70, 71]. Moreover, for HBsAg chronic hepatitis, the guidelines recommend the use of entecavir when HBV-DNA is more than 2000 IU/ml, while lamivudine is adequate at less than 2000 IU/ml. In addition, in HBV-DNA-positive cases, it may be necessary to screen for YMDD mutations in advance as there is a possibility in these cases that other treatments will be necessary, such as the use of tenofovir or the combined use of 2 nucleoside analogs [70–73].

## 5. Anti-HBc-Positive, Anti-HBs-Negative, and HBsAg-Negative Cases

Anti-HBc-positive cases indicate a prior history of HBV infection. These include window period cases as well as cases which are HBV-DNA positive and Anti-HBs negative or HBsAg negative (occult HBV infection) [43, 74–76]. Caution is required in these cases when using rituximab, chemotherapy with anticancer drugs, or immunosuppressive drug preparations [70, 71]. Although rare, HBV reactivation during chemotherapy has been reported in Anti-HBs-positive, Anti-HBc-positive cases or in cases positive only for Anti-HBc [15, 26, 43, 45]. Moreover, in a study by Hui et al., HBV reactivation-induced hepatitis was reported in anti-HBc-positive, anti-HBs-negative cases, and even in those that were HBV-DNA negative [43]. This shows that hepatitis caused by HBV reactivation can occur in Anti-HBc-positive cases regardless of HBV-DNA status. For these cases, the guidelines recommend careful monitoring of HBV-DNA, since hepatitis caused by HBV reactivation is uncommon and treatment is expensive [70–73, 77]. Although reports to date show that HBV reactivation in patients undergoing chemotherapy is rare, when it does occur, the chemotherapy treatment period is extended. In addition, the treatment intensity of the chemotherapy against lymphoma declines, and fatalities are not uncommon in HBV reactivation-induced hepatitis. This is because the mortality of hepatitis caused by HBV reactivation is about 30%–100%, and once the disease is present, some patients are not able to be saved [43–45]. Based on these facts, administration of nucleoside analogs may be desired in these cases. It has been reported, however, that HBV reactivation-related hepatitis can be preventable by monitoring HBV-DNA level on a monthly basis [43]. Further studies are needed, including calculations of the cost involved in these treatments.

## 6. Anti-HBs-Positive, Anti-HBc-Positive, and HBsAg-Negative Cases

HBV reactivation-induced hepatitis has been reported in Anti-HBs-positive, Anti-HBc-positive, HBsAg-negative cases [15, 26, 43, 45]. In addition, while rare, there have been sporadic reports of cases in which HBV reactivation was observed after administration of rituximab in patients that were positive for Anti-HBs alone [29, 43, 78] although it is generally thought that such occurrences are primarily due to vaccine administration. Such cases may arise when, for instance, the production of antibodies has declined with age and only Anti-HBs remains; however, details from these types of cases are unavailable. Caution is required, as even in cases that are positive for Anti-HBs alone, the chance exists that symptoms are due to HBV reactivation. In our experience studying the changes in Anti-HBs after rituximab administration, we have found that in the large majority of cases, Anti-HBs antibody titers decreased with increased number of rituximab administrations [21, 32, 33, 79]. Furthermore, we have experienced cases which presented with HBV reactivation following an increase in HBV-DNA that accompanied a decline in Anti-HBs and Anti-HBc antibody titers [32, 33]. These results suggest an association between Anti-HBs/Anti-HBc antibodies and HBV reactivation, and that monitoring these antibodies may provide an index for HBV reactivation. In addition, regarding HBV reactivation during bone marrow transplantation, Onozawa et al. reported that a decrease in Anti-HBs titer results in HBV reactivation-induced hepatitis [80]. As Anti-HBs is a part of the humoral immune component which monitors HBV, we surmise that transitions in Anti-HBs have the potential to become indices for predicting hepatitis caused by HBV reactivation. However, HBV reactivation has also been observed by Westhoff et al., in a patient with Anti-HBs titers of 868 mIU/mL, indicating that HBV reactivation may occur even in cases with high Anti-HBs titers [26]. This may indicate that predicting reactivation only by monitoring Anti-HBs titers would be insufficient.

## 7. Prevention of HBV Reactivation

From each group, treatment guidelines for prevention of HBV reactivation are shown in Table 1 [70–73, 77]. From the information detailed above, HBe-Ag- and HBsAg-positive cases qualify as targets for preventive administration of nucleoside analogs. In HBsAg-negative cases, it is likely that the frequency of HBV reactivation in Anti-HBc only positive cases is not necessarily high. However, since HBV reactivation may lead to patient death, we believe that these cases would qualify as targets for preventive administration of nucleoside analogs. In contrast, in HBsAg-negative, Anti-HBs-positive cases, it is desirable while observing changes in antibody titers to additionally monitor HBV-DNA in those cases with titers less than 500 [79]. Additionally, it is possible that periodic monitoring of HBV-DNA may predict HBV reactivation, and it is therefore advantageous, keeping cost in mind, to combine these indices [81, 82]. Monitoring of HBV-DNA is also likely to be essential in cases having HBV-DNA mutations and in whom antibody expression is weak [81]. It is crucial, however, to stratify groups into those in whom preventive administration can be recommended, since with HBV-DNA monitoring alone, the frequency of HBV reactivation has been reported to be higher in those given preventive administration of lamivudine compared to controls [51]. When monitoring for HBV reactivation, it is important to identify HBV reactivation at an early stage using, in addition to HBV-DNA monitoring, a variety of additional information including changes in anti-HB titers. Kusumoto et al. summarized the risks of HBV reactivation during chemotherapy in reference to HBsAg, Anti-HB, and level of treatment. These data may be useful for preventive treatment, however, more clinical studies are needed [83]. Preventive administration against HBV is recommended for a period of 6 months following completion of treatment [67, 70]. This may not be sufficient, however, as a number of cases in which HBV reactivation occurred more than 4–6 months after treatment have been reported [84–88]. The 6-month timeframe is possibly associated with changes in B-cell number [1–3, 82, 88]. The guidelines proposed by Lok and McMahon in 2007 have been more specific compared to past guidelines and have incorporated recommendations such as extending the period of preventive administration depending on the HBV-DNA level [70]. We observed a case in which HBV reactivation occurred 4 years after termination of preventive lamivudine administration following completion of treatment [79]. From this case, we surmise that for HBeAg- or HBsAg-positive cases in which Anti-HBs cannot be produced, it is necessary to combine the use of nucleoside analogs from the initial time of treatment and to continue their use. In addition, for cases which are Anti-HBs positive from the outset, we believe that preventive administration of nucleoside analogs is necessary at least until Anti-HBs titers return to pretreatment levels, and that the period of nucleoside analog administration needs to be determined using immune recovery, for example, of anti-HBs, as an index. However, with long-term administration of drugs such as lamivudine or entecavir, issues such as cost arise; therefore, it is hoped that cases requiring long-term preventive administration will become clarified in the future with followup studies. As this report shows, preventive nucleoside analog administration involves issues of lamivudine resistance, therefore we believe it is desirable to administer entecavir at an early stage. Although we commonly set lamivudine dosing at 100 mg and entecavir at 0.5 mg, it is recommended that entecavir be increased to 1 mg when dealing with lamivudine-resistant cases [70–73]. Compared to lamivudine, resistance to entecavir is less likely to develop [89], and administration of entecavir is recommended in cases where preventive administration against HBV will exceed 12 months [70, 71, 73]. In lamivudine-resistant cases, some clinicians recommend the combined use of entecavir, adefovir, or tenofovir with lamivudine [73] while others recommend switching over completely [72]. We believe that combined use with lamivudine is desirable, as it has been reported that adefovir-resistant strains immediately develop when patients with lamivudine-resistant strains are switched over to adefovir alone [90]. In seronegative HBV cases that are treated with immunosuppressive or anticancer drugs, the use of HBV vaccine has been recommended [73]. However, as mentioned previously, when using rituximab, anti-HBs level decreases and then disappears, and it is therefore possible that no antibodies will be produced after prior administration of vaccine; therefore, it may be desirable to administer vaccine after completion of treatment. In some cases, however, hepatitis caused by HBV reactivation cannot be controlled with a vaccine, and it is therefore possible that HBV reactivation will not be prevented with vaccination alone [78]. Future studies on vaccine efficacy in patients receiving rituximab or chemotherapy are needed; however, it will take considerable time before practical applications of these studies can be realized.

## 8. Conclusions

HBV reactivation during chemotherapy treatment is thought to occur from HBV amplification in hepatocytes as a result of immunosuppression caused by anticancer drugs, and subsequently from HBV-infected hepatocytes that are targeted by an immune response that becomes activated after removal of this immunosuppression [45]. It is likely that one of the causes of HBV amplification during the combined use of chemotherapy and rituximab is the reduction in antibody titers that accompanies a decrease in B cells [79, 91]. In addition, rituximab is able to alter the T lymphocyte population, and it is possible that aggressiveness toward HBV intensifies as a result, further promoting HBV amplification and subsequent recovery of the distribution of immunocompetent cells such as lymphocytes [92]. Uemura et al. found that chemotherapy-induced reactivation of HBV is more lethal compared to acute HBV hepatitis, and the likelihood of survival also decreases; therefore, it is crucial to prevent this method of disease onset [36, 44]. It is hoped that future studies will further clarify the changes that occur in immune functions of the organism during rituximab use along with the mechanisms of HBV reactivation, which will lead to safer and more effective treatments for malignant lymphomas.

## Figures and Tables

**Table 1:** Treatment guideline methods during chemotherapy or immunosuppressive drug therapy.

AASLD	Subject to preventive treatment if HBsAg positive or Anti-HBc positive & HBV-DNA positive. If HBV-DNA is less than 2000 IU, or for shortened treatment (~1 year), lamivudine or telbivudine is desirable. If HBV-DNA is more than 2000 IU and long-term treatment is necessary, entecavir or tenofovir is desirable. If HBV-DNA level remains less than 2000 IU 6 months after completion of treatment, treatment is discontinued, otherwise treatment continues (2009)
APASL	There are no guidelines (2005)

EASL	HBsAg cases are subject to treatment and HBV-DNA is to be measured in these cases although there is no defined value in which recommendation for treatment can be made. Lamivudine is most commonly used, however, it is best to be used in cases with low HBV-DNA or in conditions where resistant strains are less likely to emerge. In cases with high HBV-DNA or having a high risk of resistance, entecavir is desirable. Careful followup for HBV-DNA and liver function is necessary for HBsAG-negative, Anti-HBc-positive, and HBV-DNA-negative cases. Vaccination is recommended in HBV-seronegative cases (2009)

JAPAN	Subject to nucleoside analog treatment if HBsAg positive or if HBsAg negative and Anti-HBs or HBc positive plus HBV-DNA positive. lf HBV-DNA is negative, HBV-DNA is monitored monthly, and nucleoside analogs are administered when HBV-DNA becomes positive. Entecavir is recommended as the nucleoside analog. The timing of termination of nucleoside analog treatment will be determined in accordance to the treatment for Type B chronic hepatitis if HBsAg is positive. If Anti-HBs or Anti-HBc is positive, nucleoside analog is administered for 12 months after the completion of immunosuppressive therapy or chemotherapy. During this time, nucleoside analog treatment will be discontinued if HBV-DNA is negative and ALT is normal. Patients are closely observed for 12 months after treatment with nucleoside analogs (2009)

Abbreviations; HBsAG = hepatitis B surface antigen. Anti-HBs = antibody to HBsAg. Anti-HBc = antibody to hepatitis B core antigen. HBV-DNA = hepatitis B virus DNA.
